# Experimental Investigations on the Properties of Epoxy-Resin-Bonded Cement Concrete Containing Sea Sand for Use in Unreinforced Concrete Applications

**DOI:** 10.3390/ma12040645

**Published:** 2019-02-20

**Authors:** Sakthieswaran Natarajan, Nagendran Neelakanda Pillai, Sophia Murugan

**Affiliations:** 1 Department of Civil Engineering, Anna University Regional Campus, Tirunelveli, Tirunelveli 627007, India; sophiavarshini1992@gmail.com; 2 Department of Civil Engineering, Rajas International Institute of Technology for Women, Nagercoil 629001, India; rishi2nagendran@gmail.com

**Keywords:** sea sand, polymer concrete, mechanical strength, durability, microstructure studies

## Abstract

This paper deals with the experimental studies conducted on the effects of using sea sand on the properties of polymer concrete modified using epoxy resin. The physical properties including workability, mechanical properties, and durability properties were evaluated as a function of sea-sand substitution. The results obtained behave as strong evidence for the feasibility of using sea sand as fine aggregate to solve the problem associated with the exhaustion of natural aggregates when used in combination with epoxy polymer. A clear understanding of the behavior of polymer concrete with sea sand as aggregate was obtained through some preliminary investigations. The test results showed a significant improvement in the compressive and flexural strength due to the sea-sand substitution in polymer concrete. Resistance to the water intrusion was also improved for the concrete mixes due to the inclusion of epoxy resin. The quality and the integrity of the concrete were also improved, as evident from the SEM analysis and infrared (IR) spectroscopy, and the results function as solid basis for the use of sea-sand polymer-modified concrete for practical applications. Results also show that 15% replacement of fine aggregate by sea sand in air-cured polymer concrete exhibited enhanced strength and durability properties; thus, the produced concrete can be an effective material for unreinforced concrete applications.

## 1. Introduction

Polymer concrete is in no way new to civil engineering, and several attractive properties were achieved due to its superior performance [1]. Several polymer additives such as latex, natural rubbers, epoxy resins, and thermo-plastic polymers are added to normal concrete to produce chemically resistant, impermeable, and durable concrete [2]. Generally, polymer concrete is produced by using polymer as a partial substitute for cement [3,4]. Polymer concretes are mainly used in areas where durability is a major concern [5,6,7]. Recent years saw more attention paid to the use of sea sand as fine aggregate in concrete due to the problem of shortage of river sand [8]. Several countries almost banned the mining of river sands to protect their ecology and environment. Recent scientific technologies are aimed to utilize sea sand as a partial or complete substitute for fine aggregate, and efforts are being done to implement the process for practical applications [9]. The unstoppable grabbing of sand from the river beds can only be stopped when a perfect and feasible substitute for fine aggregate is obtained [10]. Several studies proposed to use sea-sand concrete for particular applications to enable the usage of sea-sand concrete for worldwide construction [11]. Although sea water and sea-sand concretes offer several economic and environmental benefits, they suffer from a serious drawback of corrosion when conventional reinforcements are provided [12]. The high level of chloride ions present in sea sand corrodes the traditional carbon steel, causing excessive cracking and spalling, leading to severe issues [13]. The study of chloride ion penetration forms an essential part of the durability study of sea-sand concrete, and several studies tried to analyze the influence of chloride ions on the properties of sea-sand concrete. Studies also showed that the chloride content affected the strength of sea-sand concrete, and showed that the compressive strength varied with increasing chloride concentration in the sea sand [14]. The influence of sea sand and recycled aggregates on the concrete properties showed improved strength, whereas the deformation properties decreased slightly [15]. Sea sand suffers from the severe disadvantage due to the high amount of chloride content which seems impossible to be used in reinforced concrete structure. Hence, the use of sea sand in concretes is possible only where reinforcements are not needed or when special reinforcements are provided [16]. Fiber-reinforced polymers showed the best durability performance when used as reinforcements in sea water and sea-sand concrete [17,18,19]. The modification of the property of sea sand is essential to minimize the abundance of chloride ions, which affects the durability performance of sea-sand concrete [20]. To attain maximum durability of sea-sand concrete, several admixtures such as fly ash and fumed silica were used to match with the conventional concrete [21,22,23]. Thus, the use of sea sand in concrete can be popularized only by preventing the chloride ions from destroying the passivating layer of steel surface, thereby mitigating the steel corrosion [24,25]. Several admixtures and pozzolanic additives were also tried to mitigate the dissolving of the chloride salts of sea sand and also yielded positive results [26]. A review of recent literature works showed sufficient studies on polymer concrete and sea-sand concrete [27,28,29]; however, no study attempted to test the effect of simultaneously using polymer and sea sand on the properties of concrete.

Hence, the present research work aimed at investigating the properties of epoxy-modified sea-sand concrete, as well as the internal microstructure of the produced concrete. Polymer concrete shows significant resistance to chloride penetration, and this study aimed at utilizing the lower chloride penetration capacity of epoxy concrete to mitigate the drawback of sea-sand concrete. Thus, the present work not only focuses on studying the strength variations, but also serves as an initiative to propose the widespread usage of epoxy-modified sea-sand polymer concrete to ensure sustainability in the building construction industry.

## 2. Materials

**Figure 1:**
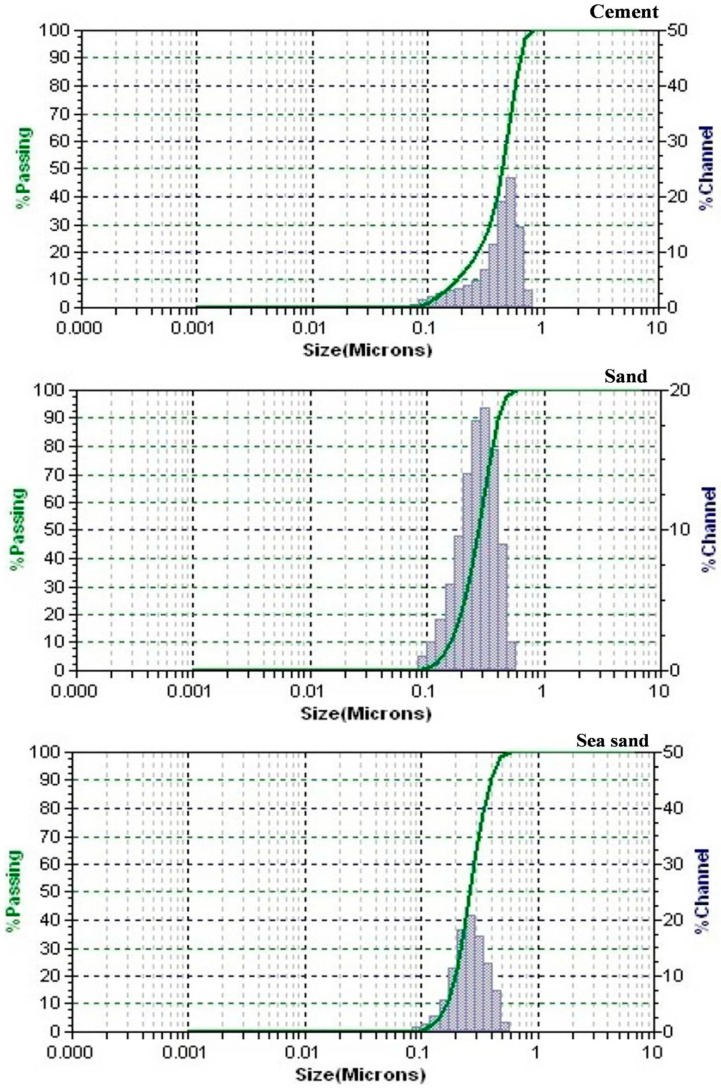
Particle size distribution of the materials used in the concrete.

Type Iordinary Portland cement (OPC) (Coramandel Cements, Hyderabad, India) was the cement used in the present study. The concrete mixes were produced using natural river sand and sea sand for fine aggregates, and coarse aggregate. The natural river sand conforms to Zone III as per BIS 383-1970 [30]. The fineness modulus of sand was 2.49 and the specific gravity was 2.58. The sea sand was collected from the nearby coastal regions (Tamil Nadu, India). The sea sand was not subjected to any treatments in the present study. Gravel of size 20mm available locally was used as coarse aggregate. Sieve analysis was done for the sample aggregate and satisfied the requirements of BIS 383-1989 [30]. The chemical characteristics of the cement and fine aggregates are shown in Table 1. The chemical composition of the cement meets the requirements of an ordinary Portland cement. The chemical composition of the natural river sand shows silica as the principal component (~99%) with minute impurities, whereas the sea sand contains only 58% silica and several oxide impurities. The presence of these impurities indicates that the sea sand was not subjected to any treatments. The gradation curve of cement and fine aggregates is presented in Figure 1. The particle size distribution curves show the uniform distribution of the aggregates with minimum fine particles, thereby exhibiting well-distributed gradation curves. Moreover, the gradation curve clearly shows the increased fineness of the sea sand when compared to the river sand. A commercial epoxy resin with the chemical base Bisphenol-A with an aliphatic hardener (Astra Chemicals, Chennai, India) was used as a polymer. M30 grade concrete as per Indian Standard method IS 10262 [31] was used to design the concrete mix. Six types of concrete mixes were produced with varying proportions of epoxy resin as a substitution for cement (2.5%, 5%, 7.5%, 10%, 12.5%, and 15%) with varying sea-sand proportion (5%, 10%, 15%, 20%, 25%, and 30%) by weight. The ordinary concrete was produced with a water–cement ratio of 0.38 with the addition of 0.75% naphthalene-based superplasticizer to attain a slump of 70 ± 2 mm. The composition of the concrete mixes showing the proportion of sea-sand substitution is summarized in Table 2, and the general proportion of the M30 grade concrete mix for 1m^3^ of the concrete is presented in Table 3.

## 3. Methodology

The workability of the polymer concrete was assessed by measuring the slump values at constant water-binder ratios (IS 1199-1959) [32]. The compressive strength of the concrete cubes of size 150 × 150 × 150 mm was determined after 14, 28, 56, 90, and 180 days (using a computer-controlled standard 400-ton-capacity compression testing machine) as per IS 516-1959 [33]. The flexural test was conducted on 100 × 100 × 500 mm beams after 28, 56, 90, and 180 days as per the standard IS 516-1959 [33], and the bond strength was attained after 28 days from the cubic specimens of size 150 × 150 × 150 mm (using an Automatic Universal Testing Machine) as per the procedure stated in ASTM C 900-15 [34]. The bulk density of the hardened concrete was obtained from the ratio of their corresponding weights and volumes as per the standard ASTM C 1754-12 [35]. The percentage of water absorption and pore spaces available in the concrete was determined as per the procedure stated in ASTM C 642-06 [36]. The rate of absorbance of concrete mixes was determined from the sorptivity tests as per ASTM C 1585-04 [37]. Fourier-transform infrared (FTIR) studies were performed on powdered concrete specimens using an Infrared Spectrometer (IR Tracer-100, Shimadzu, Japan) with KBr methodology. The scanning electron microscopic (SEM) studies of powdered concrete samples were performed using a scanning electron microscope (Philips XL20, Accretech, Austin, TX, USA). The powders were obtained from the air-cured concrete specimens and specimens that were first cured in water and then oven-dried at 105 °C for 24 h. The concrete specimens after casting were maintained at room temperature for 24 h. The concrete specimens were then demolded and were subjected to two curing conditions, namely air-curing (25 °C and 90% relative humidity (RH)) and water-curing, until the specimens were tested.

## 4. Results and discussions

### 4.1. Slump

**Figure 2:**
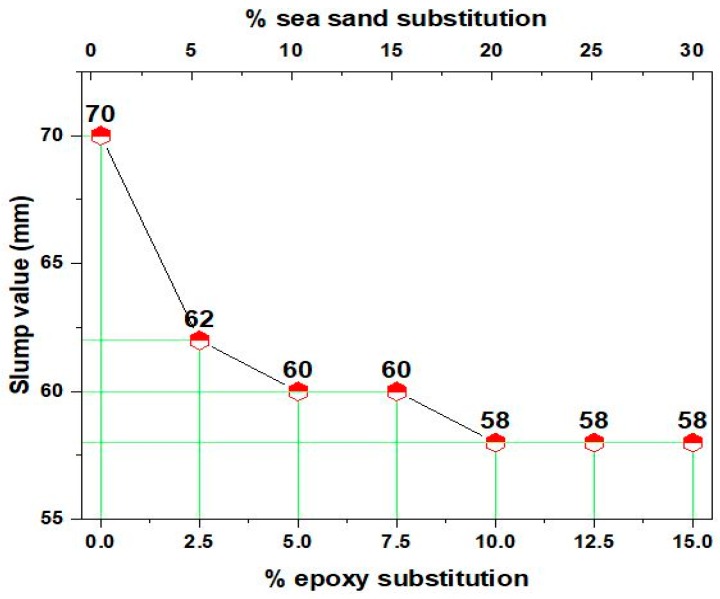
Slump values of various concrete mixes.

The workability of the epoxy-modified concrete containing different proportions of sea sand is shown in Figure 2. The slump value of the polymer concrete decreased when compared to the normal concrete. This may be due to the effect of highly viscous epoxy resin that held the concrete ingredients together, thereby reducing the slump value. This is in line with a previous study which showed that increasing content of seasand reduces the workability of concrete [22]. Generally, the substitution of sea sand as fine aggregate shows a negligible effect on the slump values of concrete [23]. The workability of fresh concrete may also be reduced with an increase in the sea-sand substitution as obtained from the experimental values. This may be due to the presence of certain amounts of minute impurities that significantly affect the workability of the concrete. A previous study showed that the presence of impurities such as coal, chalk, or clay in the sea sand does not significantly affect the concrete workability [26]. However, the workability of the presently studied sea-sand concrete was mainly dependent on the polymer concentration, which was responsible for the minute variations in the slump values of the produced polymer sea-sand concrete.

### 4.2. Bulk Density

**Figure 3:**
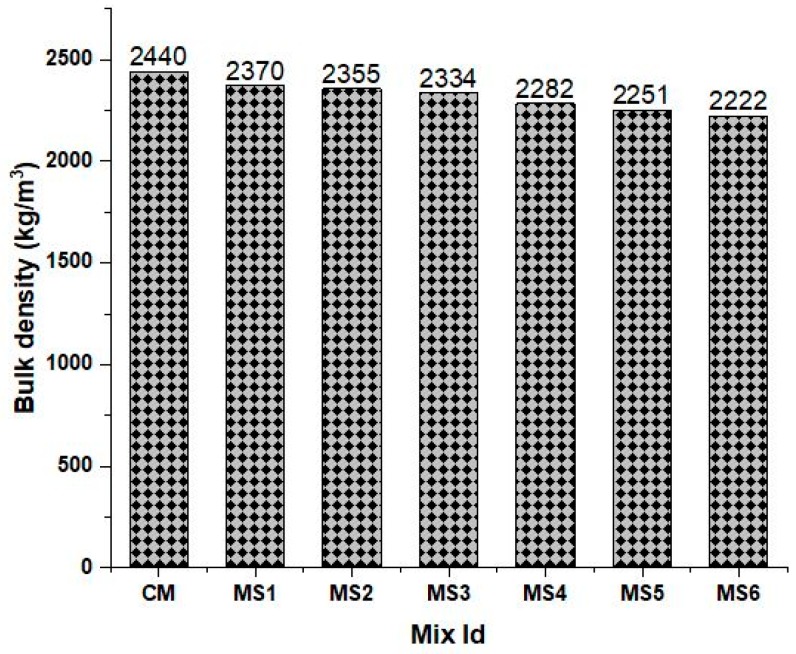
Bulk density of various concrete mixes.

The bulk densities of the concrete mixes are shown in Figure 3. The results show that the bulk density of the concrete mixes decreased slightly with increase in sea-sand substitution. However, no significant differences could be accounted for in the density variations due to epoxy substitution. The slight decrease may be due to the replacement of heavier particles with relatively lighter particles. The bulk density ranged from 2370 kg/m^3^ to 2222 kg/m^3^ with increasing sea-sand substitution ratio and epoxy substitution in the concrete.

### 4.3. Compressive Strength

**Figure 4:**
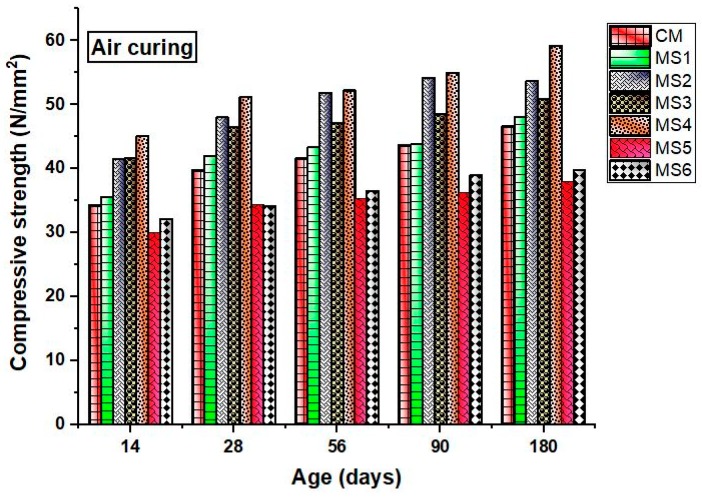
Compressive strength of the concrete mixes in air curing at various ages.

**Figure 5:**
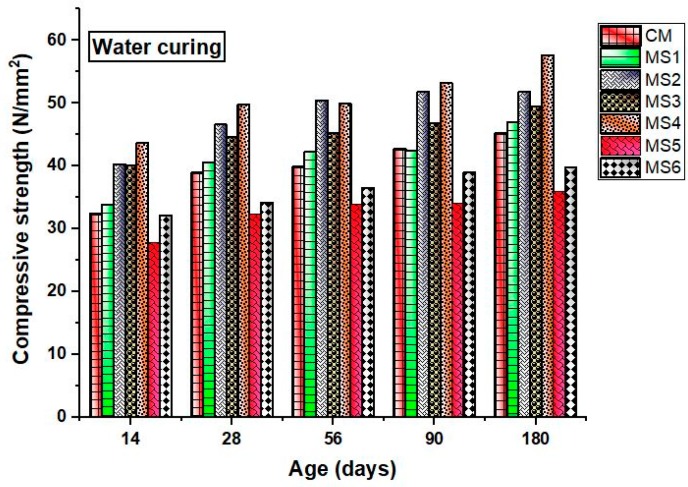
Compressive strength of the concrete mixes in water curing at various ages.

The compressive strength of the concrete specimens at various ages after being subjected to different curing conditions is shown in Figure 4 and Figure 5. Generally, the water-cured specimen exhibits higher strength when compared to the air-cured concrete specimens. On the contrary, the obtained experimental results showed improved compressive strength for the air-cured specimens rather than the water-cured specimens. The strength of the polymer concrete mixes increased linearly up to a certain percentage of sea-sand substitution, beyond which a decrease in the compressive strength was observed. The early-age compressive strength was significantly lower in water-cured specimens. Previous studies saw a severe reduction in the compressive strength due to epoxy substitution [14]. However, the present study saw an unexpected increasing trend in the compressive strength values. This may be due to the efforts taken to produce high-quality concrete mixes with proper compaction and careful proportioning.

The compressive strength of sea-sand-substituted polymer concretes showed a strength improvement in normal curing and water curing. Generally, the reaction of hydrated pastes of cement with dissolved chlorides and sulfate present in the sea sand leads to the formation of calcium bicarbonates and gypsum, leading to leaching and weakening of the insoluble aggregates. Cement reacts with dissolved salts, leading to the formation of gypsum and complex compounds like ettringite and brucite, which lead to brittleness of the cement matrix, causing strength loss and disruptive expansion. The addition of polymer retards the formation of these complex compounds by controlling the formation of Ca(OH)_2_, which is a major hydration product of cement. This helps in the formation of C_3_A (tricalcium aluminate), which enhances the durability and strength of concrete. Due to the pore-occupying nature of the polymers, they occupy the capillary pores, contributing to an increase in strength.

Thus, it can be concluded that the addition of epoxy polymer played an important role in the improvement of compressive strength at all ages. The higher strength of air-cured specimens may be due to the minimal quantity of epoxy used. An excess quantity of epoxy resins may disturb the hydration mechanism, thereby reducing the compressive strength of concrete. Sea sands have the ability to increase the compressive strength of concrete at early ages, and epoxy resin increases the strength at later ages. Previous works also saw improved compressive strength of the sea-sand concrete at early ages when compared to the strength of ordinary concrete [15].

### 4.4. Flexural Strength

**Figure 6:**
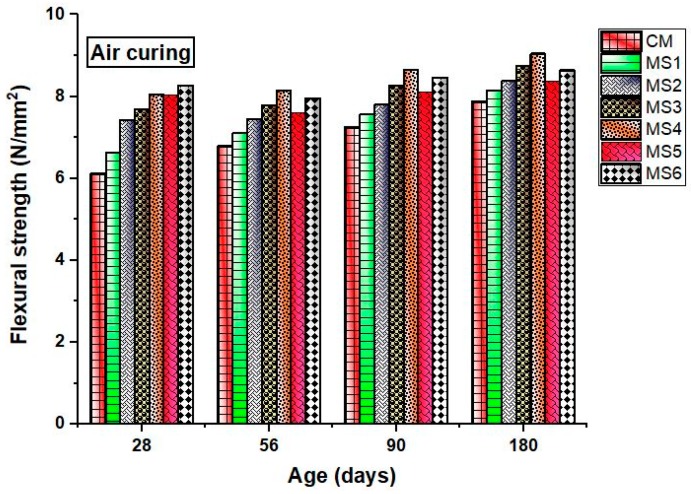
Flexural strength of the concrete mixes in air curing at various ages.

**Figure 7:**
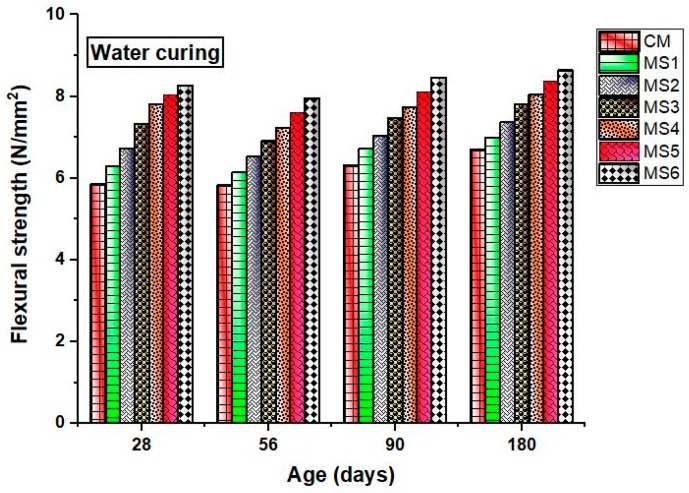
Flexural strength of the concrete mixes in water curing at various ages.

As per the flexural strength results shown in Figure 6 and Figure 7, the flexural strength of the epoxy-modified concrete increased with an increase in the sea-sand substitution at different ages when compared to the ordinary concrete. The results, thus, reveal that epoxy resin has a significant influence on improving the flexural strength of the concrete despite the increasing sea-sand substitution ratio. Results also show that sea-sand concrete exhibits better flexural performance than the conventional concrete. Studies reported that the sea sand does not reduce the flexural performance of the concrete [14]. The rate of attachment of flexural strength increased at later ages for the polymer concrete due to the effect of hardened epoxy resin. Sea-sand substitution also increases the flexural strength due to the angular surface texture and particle size acting as fillers in the epoxy resin. The reinforcing effect and filler effect of sea sand together improved the flexural strength of the concrete. The most influencing parameter was the epoxy substitution that reduced the crack formation and transformed a brittle concrete to a ductile material. Epoxy resin, which is one of the best polymeric materials, functions as a strength-contributing agent by filling the voids and retarding the ingestion of harmful ions caused due to the exchange of salts present in sea sand.

### 4.5. Bond Strength

**Figure 8:**
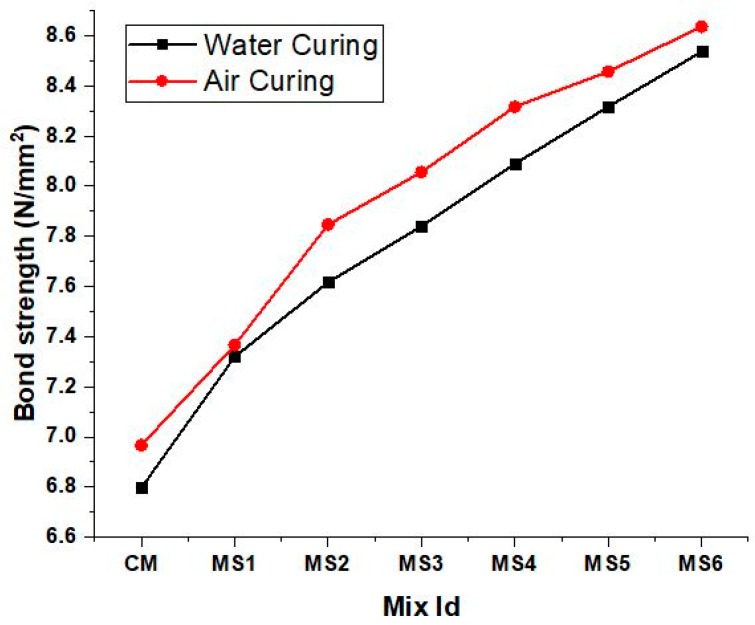
Bond strength of the concrete mixes in air and water curing.

The bond strength of the concrete mixes is shown in Figure 8. The increase in the strength of the polymer concrete may be due to the adhesive force exhibited by the epoxy polymer on the surface of the rod that created high mechanical bonding [13]. The bond strength was increased by about 25% and 23% compared to the normal concrete when the sea-sand substitution was about 15% in the air-cured and water-cured polymer concrete, respectively. The higher fineness and relatively larger surface area of the sea sand also functioned as inert fillers by effectively bonding the rod with the concrete. The interfacial transition zone has a dominant role to play in the bond strength of concrete. The epoxy resin functioned as an adhesive layer around the aggregate and also around the steel rods, thereby bonding the rod with the cement matrix.

### 4.6. Water Absorption

**Figure 9:**
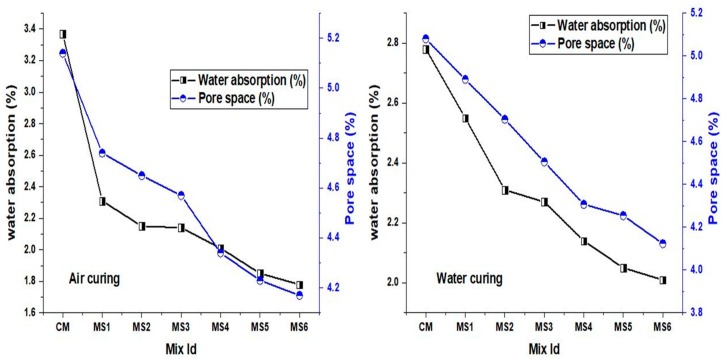
Water absorption and pore space of the air cured and water cured polymer concrete mixes.

The water absorption of the polymer concrete mixes is shown in Figure 9. A significant reduction in the water absorption values may be contributed by the epoxy resin which filled the pores in the concrete. In addition, the sea-sand substitution occupied the minute pores that are easily accessible to water. The discontinuity in the pores due to epoxy substitution and the pore-blocking capacity of the sea sand proves to be beneficial in reducing the water absorption of the concrete mixes. About a 29% reduction in the pore space was achieved in the polymer sea-sand concrete when compared to the normal concrete. The ability of the resin to form thin layers around the aggregates also reduced the micro voids in the concrete, which caused a remarkable reduction in water absorption. The water absorbed by the concrete mixes was reduced by about 48% and 42% when compared to the normal concrete when the fine aggregate was replaced by sea sand at 15% in the air-cured and water-cured polymer concrete, respectively.

### 4.7. Rate of Absorption

**Figure 10:**
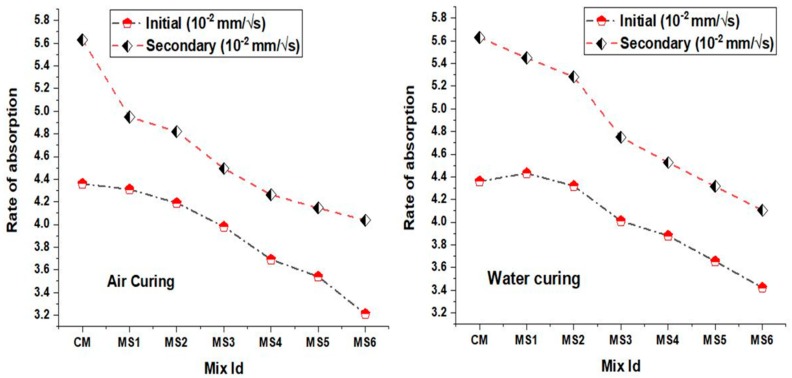
Rate of absorption of the air cured and water cured polymer concrete mixes.

Figure 10 summarizes the water absorption rate obtained from the weights of capillary water absorption at different time intervals. The initial rate of absorption corresponds to the slope of the values obtained during initial times of immersion, whereas the secondary rate corresponds to the cumulative amount of water absorbed. The rate of absorption of water is an inherent surface property of concrete which is seen to improve in the polymer concrete mixes. This shows that the concrete mixes due to sea-sand substitution are subjected to less attack of external agents even when exposed to most potential adverse conditions when used in combination with epoxy resin. Moreover, the hydrophobic property and filler capacity of the sea sand makes the concrete highly impermeable, thus enhancing the durability of concrete. Generally, an increase in the fine aggregate replacement adversely affects the permeability of inert concrete due to the increase in flowability. Here, the restriction in the flowability caused by the epoxy substitution reduced the permeability of the inner concrete.

### 4.8. FTIR

**Figure 11:**
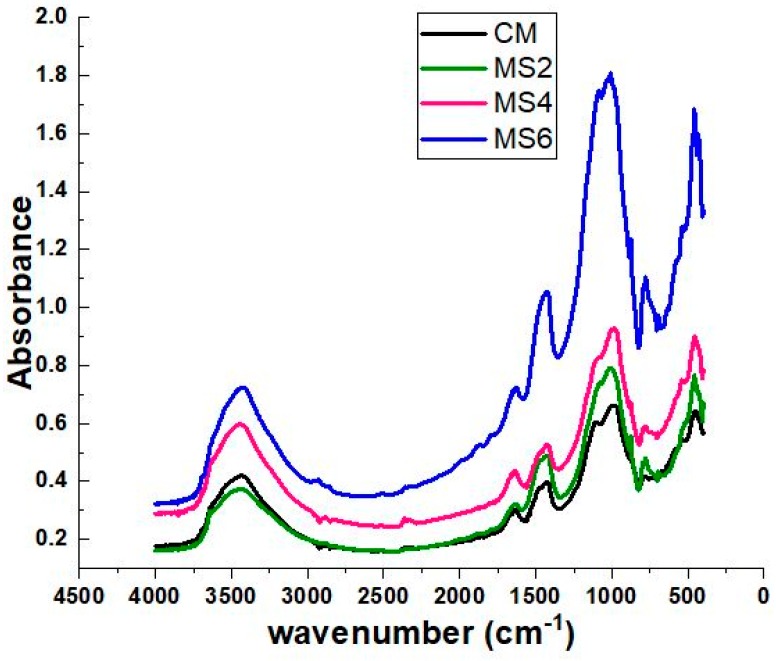
FTIR spectra of control concrete (CM), and air cured polymer concrete containing 10% (MS2), 20% (MS4), and 30% (MS6) sea sand at the age of 28 days.

FTIR spectra of the polymer concrete mixes are shown in Figure 11. The broad peak around 3400 cm^−1^ to 4000 cm^−1^ is due to the presence of –OH bonds in the concrete, which corresponds to the chemically combined water of the hydration product Ca(OH)_2_. The presence of –OH bonds in all the concrete mixes indicates the formation of well-reacted hydration products. The water crystal lattice of CSH gel may also be indicated by the –OH band. The CSH gel formation is indicated by the band formed around 1000 cm^−1^. The spectra clearly show a strong CSH band with relatively higher intensity in all the epoxy-modified sea-sand concrete mixes when compared to the normal concrete mix. The epoxy-modified concrete showed out-of-plane bending of the phenyl ring around 300–700 cm^−1^. The band around 1200cm^−1^ corresponds to the H–O–H deformation which is different in the epoxy concrete, signifying the presence of stretching vibrations of C=C of epoxy polymer. This further confirms that the epoxy resin reacted well with the H–O–H deformations to form highly stable complexes.

### 4.9. SEM

**Figure 12:**
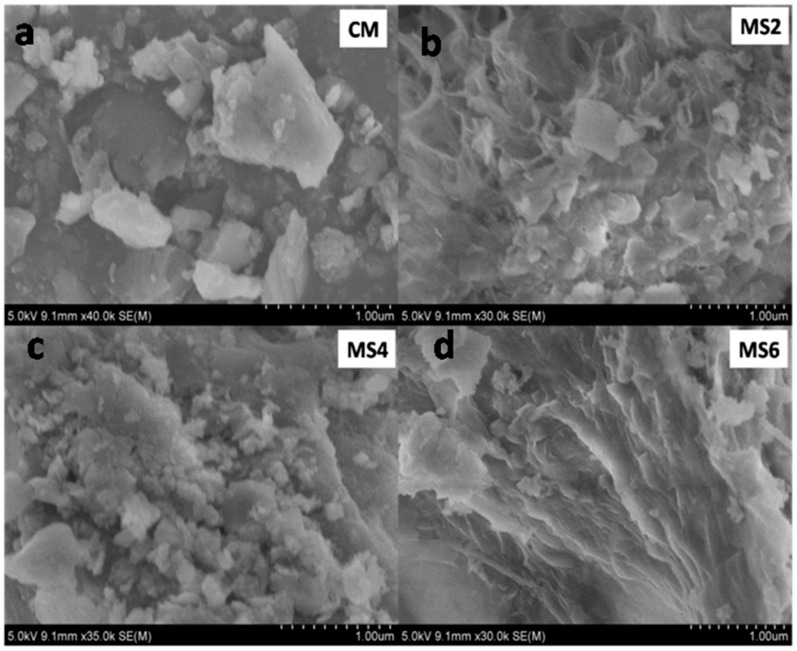
SEM images of (**a**) control concrete (CM), and air cured polymer concrete containing (**b**) 10% (MS2) (**c**) 20% (MS4), and (**d**) 30% (MS6) sea sand at the age of 28 days.

The morphology of the polymer concrete mixes and the plain concrete as obtained from the SEM imaging is shown in Figure 12. The pores or voids in concrete are indicated by the dark regions in the SEM images. No visible pores are evident from the obtained SEM images of the concrete. The pores in the concrete were absent in the polymer concrete mixes, which supports the filling capacity of sea sand. The presence of flaky gel-like structures present in the polymer concrete indicates the presence of gelatinous epoxy resin. The interfacial zone between the aggregates and the cement paste also showed a thin layer of epoxy polymer that formed an adhesive layer in the concrete, which caused a reduction in the porosity. The micro images also show the bonding between epoxy resin and sea sand, which confirms the mechanical strength results.

### 4.10. Limitations of the Present Work and Future Study

Generally, sea sand should be subjected to pre-treatments or de-salting procedures to remove the excess chloride content for use in concrete applications due to itsadverse effect on concrete properties by promoting corrosion of reinforcement. The present study utilized sea sand without appropriate de-salting procedures and, hence, cannot be used for reinforced structural applications, which is the limitation of the present study. The high cost associated with the de-salting procedure and lack of appropriate technologies prompted the direct use of sea sand in concrete. With this background, future studies will try to determine the possibility of using sea sand in epoxy-modified concrete for reinforced concrete applications by conducting corrosion studies, thereby ensuring the safety and durability of the proposed methodology.

## 5. Conclusions

In the current investigation, the properties of concrete utilizing epoxy resin as a cement substitute and sea sand as a substitute for river sand were investigated to their suitability for unreinforced concrete applications. Both epoxy substitution and sea-sand substitution affect the workability of concrete, but the effect of the latter is minimal. The compressive strength development was accelerated at the early ages due to sea sand, whereas the polymer cement concrete produced stronger concrete when compared to the conventional concrete at the latter ages. About 30% improvement in the compressive strength and 35% improvement in the flexural strength were achieved in the 20% sea-sand-substituted air-cured polymer concrete at 180 days when compared to the conventional concrete. The flexural strength of the epoxy concrete was further enhanced by the incorporation of sea sand that acted as a micro filler. Bond strength was markedly superior with improved pull-out strength of the polymer concrete in the presence of sea sand as fine aggregate. Other parameters such as water absorption and pore spaces were reduced showing the superior performance of epoxy-modified sea-sand concrete. Compared to ordinary concrete, the initial and secondary rate of water absorption was also reduced, showing the greater resistance to water permeability. The microstructure of polymer sea-sand concrete showed significant morphological improvement at the inside of the concrete, indicating the bonding between sea sand and the cement matrix. Thus, a limited supply of fine aggregates may be compensated for by the utilization of sea sand as fine aggregate without disturbing the ecosystem and sustainability of the concrete structures when used in combination with epoxy resins.

## Figures and Tables

**Table 1:** Chemical composition of the cement, sand, and sea sand.

Component	Cement (%)	River Sand (%)	Sea Sand (%)
SiO_2_	41.447	99.27	55.818
GeO_2_	–	0.721	–
Fe_2_O_3_	2.490	–	21.671
CaO	53.781	–	–
TeO_2_	–	0.009	–
K_2_O	1.087	–	–
TiO_2_	1.195	–	–
V_2_O_5_	–	–	2.520
Cr_2_O_3_	–	–	1.812
Ga_2_O_3_	–	–	2.101
Br_2_O	–	–	6.680
Re_2_O_7_	–	–	9.398

**Table 2:** Composition of the concrete mixes showing the proportion of ingredients (% by weight).

MIX Identifier	w/b Ratio (%)	BINDER		FINE AGGREGATE		Coarse Aggregate
		CEMENT	EPOXY RESIN	RIVER SAND	SEA SAND	(20 mm Gravel)
		%	%	%	%	%
CM	0.38	100	–	100	0	100
MS1	0.38	97.5	2.5	95	5	100
MS2	0.38	95	5	90	10	100
MS3	0.38	92.5	7.5	85	15	100
MS4	0.38	90	10	80	20	100
MS5	0.38	87.5	12.5	75	25	100
MS6	0.38	85	15	70	30	100

**Table 3:** Proportion of M30 grade concrete.

Materials	Materials (kg/m^3^)	Mix Ratio
Cement	391.580	1
River sand	638.560	1.63
Gravel	1245.650	3.18
Water	148.800	0.38
Super plasticizer	2.937	0.0075
